# Effect of Hormone Replacement Therapy on Liver and Cardiometabolic Outcomes in Peri‐Menopausal MASLD

**DOI:** 10.1111/liv.70562

**Published:** 2026-02-23

**Authors:** Alex E. Henney, Jo Wilson, David R. Riley, Uazman Alam, Daniel J. Cuthbertson

**Affiliations:** ^1^ Department of Cardiovascular and Metabolic Medicine University of Liverpool Liverpool UK; ^2^ Metabolism and Nutrition Research Group, Liverpool University Hospitals NHS Foundation Trust Liverpool UK; ^3^ Liverpool Centre for Cardiovascular Sciences, University of Liverpool and Liverpool University Hospitals NHS Foundation Trust Liverpool UK; ^4^ Centre for Musculoskeletal Research, Faculty of Biology Medicine and Health The University of Manchester Manchester UK

## Abstract

**Background:**

Metabolic dysfunction‐associated steatotic liver disease (MASLD) is the most common chronic liver disease globally. Menopause is associated with increased hepatic fat deposition and thus metabolic dysfunction, contributing to heightened risk of progressive liver and cardiovascular disease. Hormone replacement therapy (HRT), supported by pre‐clinical data, may be associated with a lower risk.

**Methods:**

We performed a retrospective cohort study using the TriNetX global federated research network. Eligible participants were peri‐menopausal women (ICD‐10 codes N95/Z78.0, AND age 40–65 years) with pre‐existing MASLD (based on ICD‐10 codes K76.0/K75.81 or positive modified hepatic steatosis index plus ≥ 1 metabolic syndrome, MetS, trait). Patients initiating HRT (oestrogen ± progesterone) were compared with untreated controls using 1:1 propensity score matching for demographics, comorbidities, biochemistry and medications. The primary outcome was a composite of major adverse liver outcomes (MALO: portal hypertension, varices, ascites, spontaneous bacterial peritonitis, encephalopathy, hepatorenal/pulmonary syndromes, cirrhosis, decompensated liver disease, hepatocellular carcinoma, liver transplant). Secondary outcomes were individual MALO components, type 2 diabetes (T2D), major adverse cardiovascular events (MACE), breast and endometrial cancer, and venous thromboembolism (VTE). Cox regression generated hazard ratios (HRs) with 95% CIs over 5 years. Sensitivity analyses adjusted for geography, hormone type, and degree of obesity.

**Results:**

After matching, 21 639 patients were included in each treatment arm. HRT was associated with a significantly reduced risk of MALO (HR 0.80; 0.71, 0.9), largely driven by reductions in ascites and SBP (0.78; 0.64, 0.95), and liver cirrhosis (0.75; 0.63, 0.90), and reduced risk of cardiometabolic outcomes: T2D (0.90; 0.84, 0.96), and MACE (0.90; 0.83, 0.98). HRT was not associated with increased risk of breast cancer or VTE, whilst endometrial cancer risk was reduced (0.49; 0.40, 0.61). Oestrogen was linked to greater benefits compared to progesterone, and patients with mild–moderate obesity experienced more significant risk reduction.

**Conclusion:**

Treatment of peri‐menopausal symptoms with HRT, in patients with pre‐existing MASLD, is associated with a lower 5‐year risk of major liver and cardiometabolic disease. These findings support early basic science research and should prompt a closer examination through clinical trials.

## Key Points

- Hormone replacement therapy (HRT) use in peri‐menopausal women with metabolic dysfunction‐associated steatotic liver disease (MASLD) was associated with a lower risk of major adverse liver outcomes over 5 years.
- HRT was also linked to reduced incidence of type 2 diabetes and major adverse cardiovascular events, without increased risk of breast cancer or venous thromboembolism.
- These real‐world findings support a potential protective role for HRT in MASLD and highlight the need for prospective clinical trials to confirm causality and guide clinical practice.

## 1 Introduction

Metabolic dysfunction‐associated steatotic liver disease (MASLD) is the most common chronic liver disease worldwide, affecting ~30% of the adult population [1]. It represents a disease spectrum ranging from simple steatosis and steatohepatitis through to progressive fibrosis and cirrhosis, with an annual fibrosis progression rate of 0.9% [2]. Considering its association with hepatic and extra‐hepatic complications, such as cirrhosis, hepatocellular carcinoma and cardiovascular disease, it poses a significant global public health challenge. The prevalence of MASLD continues to increase globally, affecting diverse populations [2].

The menopausal transition represents a pivotal period of metabolic and hormonal change that directly influences hepatic and cardiometabolic health. Declining circulating oestrogen levels have been associated with adipose tissue redistribution, hepatic lipid accumulation and insulin resistance. These changes are biologically plausible contributors to MASLD progression and increased cardiovascular risk, although the extent to which hormonal changes directly drive disease progression remains uncertain. Prior to the menopause, women have a lower risk of MASLD than men, but in the post‐menopausal period, the prevalence can be similar or higher to that in men [3, 4]. Consequently, peri‐ and post‐menopausal women with MASLD could be considered a distinct subgroup at high risk of MASLD.

Lifestyle modification with increased physical activity and weight loss remains the cornerstone of management of MASLD; however, its efficacy in reversing steatosis and fibrosis is modest, with long‐term maintenance of weight loss challenging [5]. To date, pharmacological treatment options for MASLD are scarce and restricted to two agents. The thyroid hormone receptor‐β (THR‐β) agonist, resmetirom, remains restricted to the treatment of a subset of patients with non‐cirrhotic steatohepatitis and stage 2 or 3 fibrosis (regulatory approval, 2024); widespread implementation has not been achieved. More recently, semaglutide, a glucagon‐like peptide‐1 receptor agonist (GLP‐1 RAs), has also been approved [6]. For women in the menopausal transition, hormone replacement therapy (HRT), given for symptomatic relief of oestrogen deficiency, may have a beneficial effect on MASLD. Activation of oestrogen receptor‐α suppresses lipogenesis, enhances β‐oxidation via PPAR‐α, and reduces hepatic inflammation and fibrosis by limiting cytokine release and stellate‐cell activation [7]. Therefore, loss of oestrogen expectedly promotes visceral fat accumulation and inflammation; processes that drive hepatic lipid flux and fibrosis in MASLD [8]. Progesterone may exert additional or modulating effects, although these remain less well defined [9].

While pre‐clinical studies consistently demonstrate that oestrogen attenuates hepatic steatosis and fibrosis, human data examining the impact of HRT on MASLD progression are lacking. It remains unclear whether HRT modifies the natural history of MASLD by reducing the risk of major adverse liver outcomes (MALOs), such as cirrhosis or hepatic decompensation. Furthermore, the relative individual contributions of oestrogen and progesterone, and whether combination therapy offers an additive benefit, have not been elucidated. Such unanswered questions represent a significant gap in the current understanding of MASLD pathophysiology within the peri‐menopausal population; such women may derive dual hepatic and cardiometabolic benefit from HRT. Importantly, although current NICE guidance (NG23, 2024 update) recommends HRT for menopausal symptom relief and osteoporosis prevention, the potential hepatic or metabolic benefits are not yet recognised [10], reflecting a lack of robust evidence.

With this in mind, this study aimed to evaluate the impact of HRT on major adverse liver outcomes (MALO) in peri‐menopausal women with pre‐existing MASLD, and to evaluate its effects on cardiometabolic health outcomes including type 2 diabetes and major adverse cardiovascular events using large‐scale real‐world data.

## 2 Methods

### 2.1 Specification of the Target Trials

#### 2.1.1 Study Overview

We compared the outcomes of peri‐menopausal women and MASLD with new use of HRT (oestrogen and/or progesterone) versus outcomes in those not using HRT, on the time to a first‐time diagnosis of MALO, using a target trial emulation framework. Data S1 lists key protocol components. To enhance clarity, we explicitly separate the specification of the idealised target trial from its emulation in the TriNetX platform (Table S1). The target trials are specified as follows:

### 2.2 Eligibility Criteria

*Inclusion criteria* for all target trials included peri‐menopausal patients (ICD‐10 codes N95/Z78.0, AND age 40–65 years) with pre‐existing MASLD (defined using ICD‐10 code K76.0 for fatty changes to the liver, or K75.81 for non‐alcoholic steatohepatitis, or a risk profile, suggestive of hepatic steatosis; given the underdiagnosis of hepatic steatosis, we used a previously adopted and validated formula to identify additional diagnoses not detected through ICD‐10 codes [11]. This formula is a modified hepatic steatosis index (HSI), which can be calculated in the TriNetX platform. This followed that patients are considered high risk for MASLD if they: (i) are over 50 years; AND (ii) have an alanine aminotransferase ≥ 30 U/L; AND (iii) have body mass index (BMI) ≥ 30 kg/m^2^) who had medical encounters with a Health Care Organisation (HCO) and were prescribed one of oestrogen and/or progesterone.

*Exclusion criteria* included other causes of liver disease (alcohol‐related liver disease, viral hepatitis, primary sclerosing cholangitis, primary biliary cirrhosis, haemochromatosis, Wilson's disease, toxic liver disease), a diagnosis of MALO or markers of severe liver disease prior to the index event (portal hypertension, ascites, spontaneous bacterial peritonitis (SBP), hepatic encephalopathy, hepatorenal or pulmonary syndrome, oesophageal or gastric varices, liver failure, cirrhosis, hepatocellular carcinoma, liver transplant, platelets < 100 10^3^ u/L, albumin < 2.8 g/dL, bilirubin > 2 g/dL, ammonia > 50 umol/L, prescription of rifaximin, carvedilol, high dose spironolactone, or neomycin), or contraindications and limited use information for HRT (history of hormone‐dependent cancer, including breast and endometrial cancer, venous thromboembolism, including deep vein thrombosis and ischaemic stroke, and migraine with aura). Finally, the reference arm could not, at any point, be prescribed HRT. Please see Table S2 for a full list of definitions.

#### 2.2.1 Treatment Strategies

The treatment strategy included either one of initiation of HRT at baseline (index event) or diagnosis of the menopause. Patients in the reference arm could not have been prescribed HRT at any point in the electronic health record history. Initiation of use is defined as the first prescription for the drug, consistent with an intention‐to‐treat design. The treatment strategy is assigned at baseline, regardless of medication use adherence, medication switch, or add‐on. Analysis was of new starters of HRT, 1 day after drug initiation. Patients were followed up for 5 years.

#### 2.2.2 Study Outcomes

The *primary outcome* was a first‐time diagnosis of a MALO composite documented in patient electronic health records (EHRs): portal hypertension, oesophageal or gastric varices, ascites, spontaneous bacterial peritonitis, hepatic encephalopathy, hepatorenal or pulmonary syndrome, hepatic failure or cirrhosis, hepatocellular carcinoma, and liver transplant.

*Secondary outcomes* included the individual MALO endpoints, as well as a MACE composite (acute coronary syndrome, cerebrovascular accident, heart failure, peripheral vascular disease, or sudden cardiac death), and separately, T2D. We also included negative outcomes associated with HRT including breast cancer and deep vein thrombosis (Table S2, full definitions).

For each outcome analysis, individuals with prevalent disease at baseline were excluded to ensure assessment of incident events only. Specifically, for analyses of incident T2D, all patients with a recorded diagnosis of T2D prior to index date were excluded. Similarly, for analyses of incident MACE, patients with a history of MACE before index date were excluded. In contrast, for the primary MALO analysis, patients with prevalent T2D or MACE were retained and included as covariates in the propensity score model to balance baseline cardiometabolic risk between exposure groups. This outcome‐specific exclusion approach ensured appropriate risk sets for each endpoint while maintaining balanced baseline characteristics.

#### 2.2.3 Follow‐Up

**FIGURE 1:**
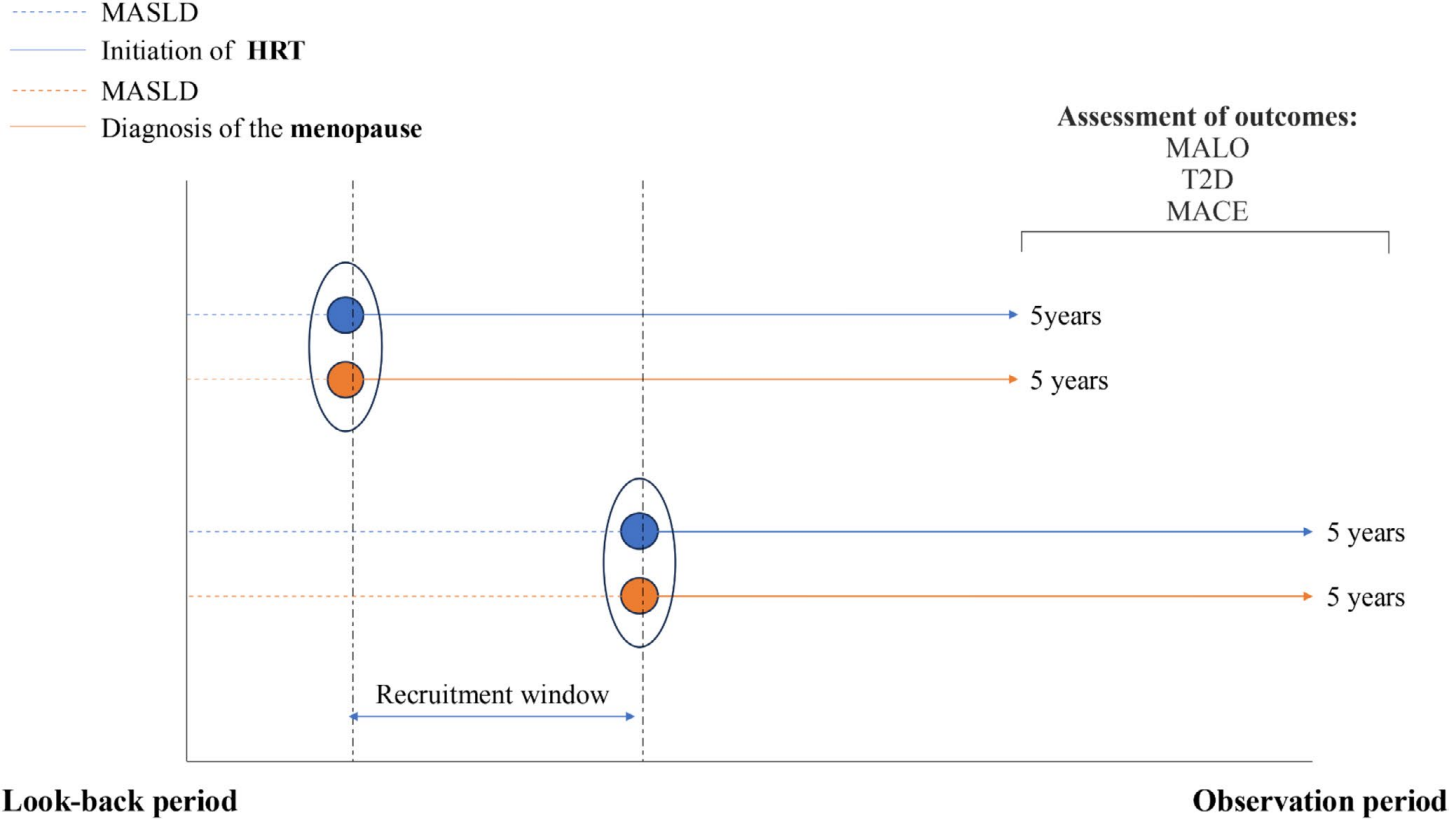
Timeline of patient recruitment to the study. Patients were followed up for a maximum of 5 years from the index event mean follow‐up time was 1099 days in the HRT arm, and 1100 days in the no HRT arm.

Each eligible patient was followed from the index event until the occurrence of the outcome, death, loss to follow‐up, or 5 years after the index event, whichever occurred first (Figure 1).

#### 2.2.4 Analysis Approach

The causal estimates represent the intention‐to‐treat effect of being assigned to different treatment strategies. Cumulative incidences were estimated using Kaplan–Meier survival analysis in propensity‐score matched (1:1 using nearest‐neighbour greedy matching, calliper 0.25 SD) patients. Hazard ratios (HRs) and 95% confidence intervals (CIs) were calculated, with models adjusted for baseline confounders via propensity‐score matching (PSM). A parallel Cox regression estimates the HR, but the “Compare Outcomes” TriNetX model isolates the effect of the index event, assuming other cohort aspects are equal. Although the user interface doesn't explicitly show stratified Cox models, PSM is performed before outcome comparison to balance baseline covariates. This ensures the HR compares well‐matched cohorts, controlling for confounding via matching rather than simultaneous adjustment for multiple covariates like in traditional Cox regression.

### 2.3 Emulation of the Target Trials

#### 2.3.1 Study Design

We explicitly emulated the target trials described previously using data and built‐in analytic functions on the TriNetX Analytics platform. TriNetX (LLC, Cambridge, MA, USA) is a global federated health research network with access to both inpatient and outpatient electronic medical records from health care organisations internationally; largely secondary and tertiary care providers in North America and Western Europe. This analysis was conducted on the Global Collaborative Network, which contains data from almost 180 million patients across around 150 HCOs, with access to diagnoses, procedures, medications, laboratory values and genomic information worldwide. Data was collected in September 2025. The built‐in analytics within the TriNetX Analytic platform analysed patient‐level data; however, only population‐level results are reported to users. TriNetX data are HIPAA (Health Insurance Portability and Accountability Act) de‐identified and access to protected health information is not allowed. Therefore, there is no risk for protected health information disclosure, and Institutional Review Board review was not required. Further details on the network have been described elsewhere [12].

Each component of the target trial was emulated using EHRs from the TriNetX Analytics platform. Patients were classified into treatment and reference arms: treatment arm (HRT) or reference arm (no HRT), based on the first prescription in the study period or diagnosis of the menopause, which was the baseline or index event. Eligibility criteria and 48 covariates were evaluated at baseline. This included a look back period set to “anytime” within the TriNetX Analytical platform; this is capped at a maximum of 20 years, and therefore the earliest date looked back to was 2005. The treatment and reference arms were separately propensity‐score matched for covariates at the baseline to emulate randomisation. After propensity‐score matching, all groups must have been considered well balanced using a standardised mean difference of < 0.1.

#### 2.3.2 Propensity Score Matching

Cohorts were PSM, in a 1:1 ratio, for (i) sociodemographic variables: age, sex, ethnicity, smoking, alcohol‐use disorder (AUD) (AUD was included as a baseline covariate in propensity score matching. However, systematic data on alcohol consumption were not available within TriNetX, and therefore patients with moderate alcohol use and thus potential MetALD could not be reliably identified or excluded), socioeconomic status (problems relating to education and literacy, employment, housing, and psychosocial circumstances), (ii) comorbidities: cardiovascular disease (ischaemic heart disease (IHD), cerebrovascular accident (CVA), hypertension and dyslipidaemia), type 2 diabetes, and thyroid disease, and cancer, (iii) anthropometrics: body mass index (BMI), (iv) biochemistry: HbA1c, platelets, albumin, alanine (ALT) and aspartate aminotransferase (AST), bilirubin and prothrombin time, and (v) medication: glucose lowering therapy, lipid‐lowering therapy, anti‐coagulation, anti‐platelets, steroids and anti‐hypertensives. All biochemical and anthropometric variables used in the cohort creation (i.e., HbA1c and BMI) must have been the most recent recorded value prior to the index event, however we cannot state the exact duration for each patient as we do not have individual level data. Definitions for all PSM covariates are presented in Table S2.

#### 2.3.3 Statistical Analysis

Statistical analysis was performed in situ within TriNetX. TriNetX uses the R Survival package v3.2‐3. Additionally, for sensitivity analysis, we: (i) performed subgroup analysis by geographical location (patients registered to HCOs in the USA, only), (ii) stratified patients by specific hormone replacement therapy type (oestrogen or progesterone), (iii) stratified patients by their degree of obesity (mild–moderate vs. severe obesity, BMI < or > 40 kg/m^2^), (iv) stratified patients by ethnicity (white vs. ethnic minority group), (v) redefined MALO by excluding portal hypertension and non‐bleeding varices (which are not true decompensation events), and by restricting variceal events to oesophageal varices with haemorrhage (ICD‐10 I85.0); the remaining MALO components were unchanged, and (vi) restricted the exposure definition to patients with evidence of persistent HRT use (defined as ≥ 2 HRT prescriptions separated by at least 6 months), thereby reducing misclassification from short‐term or transient exposure. Finally, we calculated *E*‐values, representing the minimum strength of association on the HR scale that an unmeasured confounder would need to have with both the exposure (treatment arm) and the outcome, conditional on the measured confounders, to explain away the observed association; HR + √[HR × (HR −1)] [13]. The Strengthening the Reporting of Observational Studies in Epidemiology (STROBE) guidelines were followed in the reporting of this cohort study [14].

## 3 Results

### 3.1 Study Population

**FIGURE 2:**
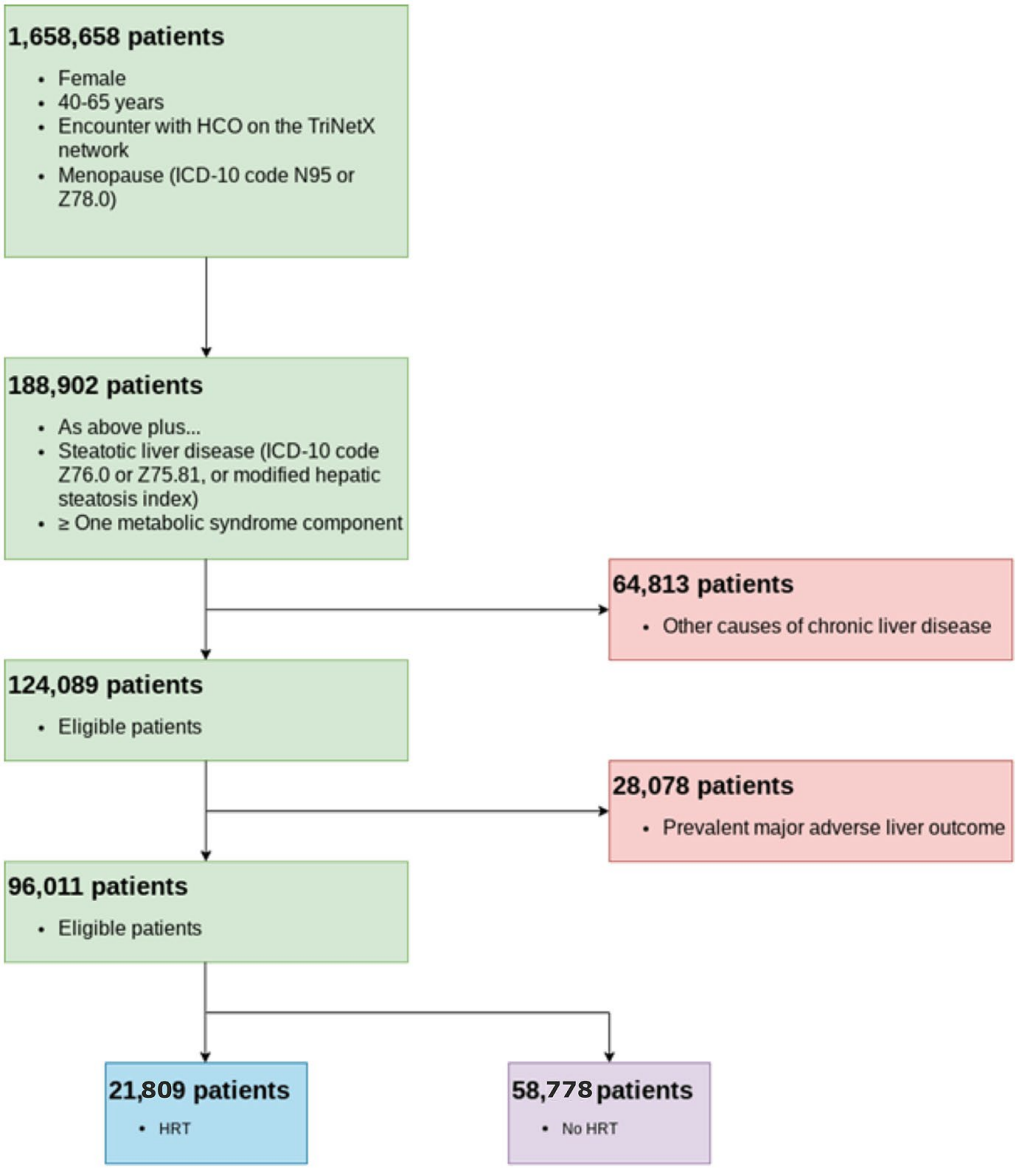
CONSORT diagram demonstrating the design of the study including the number of patients meeting inclusion and exclusion criteria.

The study timeline and a CONSORT diagram demonstrate the cohort composition and reasons for exclusions (Figures 1 and 2). The study included a total of 80 089 patients: 21 809 patients were prescribed HRT, and 58 778 patients were not (Table 1).

**TABLE 1:** Baseline covariates of patients in the HRT and no HRT group.

Characteristic	Before propensity score matching			After propensity score matching		
	HRT	No HRT	SMD	HRT	No HRT	SMD
*Demographics*						
Numbers (*n*)	21 809	58 778		21 639	21 639	
Age (years)	53 ± 6	52 ± 6	0.06	53 ± 6	53 ± 6	< 0.01
Ethnicity (%)						
White	74.4	74.5	< 0.01	74.4	74.8	0.01
Black	8.8	9.6	0.03	8.8	9.0	0.01
Hispanic	15.4	20.3	0.13	15.5	14.4	0.03
Asian	4.0	4.8	0.04	4.0	3.9	0.01
Socioeconomic hazards (%)	5.8	4.0	0.08	5.7	5.5	0.01
Nicotine dependence (%)	12.9	12.8	< 0.01	12.9	13.4	0.01
*Biochemistry (Data completeness (%))*						
HbA1c (%)	6.1 ± 1.3	6.3 ± 1.4	0.11	6.1 ± 1.3	6.1 ± 1.3	0.03
ALT (U/L)	32 ± 27	33 ± 29	0.01	32 ± 27	32 ± 29	0.01
AST (U/L)	28 ± 20	28 ± 22	0.01	28 ± 20	28 ± 20	0.01
Albumin (g/dL)	4.2 ± 0.4	4.2 ± 0.4	0.07	4.2 ± 0.4	4.2 ± 0.4	0.02
Platelets (x10^9^/L)	275 ± 70	275 ± 72	0.01	275 ± 70	276 ± 72	0.01
Bilirubin ()	0.5 ± 0.3	0.5 ± 0.3	< 0.01	0.5 ± 0.3	0.5 ± 0.4	0.02
Prothrombin time (s)	11.8 ± 3.2	11.8 ± 4.2	0.01	11.8 ± 3.2	11.9 ± 4.3	0.02
*Anthropometrics (Data completeness (%))*						
Body mass index (kg/m^2^)	33.2 ± 7.3	34.3 ± 7.7	0.14	33.2 ± 7.3	33.5 ± 7.4	0.05
*Comorbidity (%)*						
Cardiovascular disease						
Ischaemic heart disease	8.8	7.4	0.05	8.8	8.6	0.01
Cerebrovascular accident	5.1	4.3	0.04	5.1	5.2	< 0.01
Peripheral vascular disease	3.1	2.6	0.03	3.1	2.9	0.01
Heart failure	2.5	2.4	< 0.01	2.5	2.5	< 0.01
Cancer	50.7	41.9	0.18	50.5	50.0	0.01
Thyroid dysfunction	32.6	25.7	0.15	32.4	32.0	0.01
Metabolic syndrome						
Type 2 diabetes	28.1	26.2	0.04	28.0	27.6	0.01
Hypertension	49.2	46.3	0.06	49.1	48.7	0.01
Dyslipidaemia	56.1	47.6	0.17	55.9	55.7	< 0.01
*Concomitant medication (%)*						
Corticosteroids	65.6	50.8	0.30	65.5	65.5	< 0.01
Lipid lowering therapy	35.1	25.9	0.20	34.9	35.1	< 0.01
Anti‐hypertensives						
Beta blockers	28.5	22.9	0.13	28.4	28.2	0.01
ACE inhibitors	19.4	15.8	0.10	19.3	19.5	0.01
Angiotensin receptor blockers	15.0	11.2	0.11	14.9	14.5	0.01
Calcium channel blockers	15.2	12.2	0.09	15.2	15.3	< 0.01
Diuretics	26.8	21.1	0.14	26.7	26.5	0.01
Alpha blockers	5.5	3.6	0.09	5.4	5.3	< 0.01
Blood modulating agents						
Anti‐coagulation	23.7	20.5	0.08	23.7	23.7	< 0.01
Aspirin	20.0	15.6	0.12	19.9	19.7	0.01
Glucose lowering therapy						
Insulin	13.7	12.3	0.04	13.7	13.9	0.01
Tirzepatide	3.4	1.7	0.10	3.2	3.0	0.01
Semaglutide	8.7	4.7	0.16	8.4	7.9	0.02
Liraglutide	4.0	2.1	0.11	3.8	3.5	0.01
Dulaglutide	4.4	2.5	0.10	4.2	4.1	0.01
Exenatide	1.1	0.6	0.05	1.0	1.0	< 0.01
Glipizide	3.9	2.8	0.06	3.8	3.9	< 0.01
Glimepiride	2.0	1.7	0.03	2.0	2.1	< 0.01
Empagliflozin	3.6	2.3	0.08	3.5	3.5	< 0.01
Dapagliflozin	1.4	1.0	0.03	1.4	1.4	< 0.01
Canagliflozin	1.1	0.7	0.04	1.0	1.1	< 0.01
Sitagliptin	3.3	2.6	0.04	3.3	3.3	< 0.01
Linagliptin	0.7	0.6	0.01	0.7	0.8	< 0.01
Pioglitazone	1.3	1.0	0.03	1.3	1.3	< 0.01

### 3.2 Baseline Characteristics

The mean age in the two arms was similar (HRT arm, 53 years vs. control arm, 52 years) with a lower BMI and HbA1c in the HRT arm (33.2 vs. 34.3 kg/m^2^). Patients in the HRT arm were also, on average, more likely to be living with dyslipidaemia, cancer, thyroid disease, and be prescribed steroids, lipid‐lowering therapy, certain anti‐hypertensive and glucose lowering therapies, and aspirin. After PSM, each cohort was deemed well matched (Figure S1). The total number of participants in each cohort was reduced to 21 639 (Table 1).

### 3.3 Survival Analysis

The mean follow‐up time in the HRT arm was 1099 days, whereas for the no HRT arm it was 1100 days.

#### 3.3.1 Primary Outcome

Treatment of peri‐menopausal symptoms with HRT was associated with a reduced risk of MALO (HR 0.80; 95% CI 0.71, 0.90). Of the 21 639 patients included in each arm, 501 patients in the HRT arm progressed to a MALO (compared to 625 patients who didn't receive HRT), with an incidence rate of 23.1 (vs. 28.9) per 1000 person‐years.

#### 3.3.2 Secondary Outcomes

##### 3.3.2.1 Individual MALO Composites

Specifically, associated risks of ascites or SBP (0.78; 0.64, 0.95) and liver failure or cirrhosis (0.75; 0.63, 0.90) were reduced.

##### 3.3.2.2 Cardiometabolic Outcomes

From 15 274 patients in the HRT arm without prevalent T2D, 1713 progressed to T2D (compared to 1897 from 15 179 patients in those not treated with HRT). For MACE, from 20 175 patients without prevalent disease, 899 progressed to incident MACE (compared to 992 from 20 089 patients in those not treated with HRT). This corresponded to a reduced associated risk of cardiometabolic outcomes: MACE (0.90; 0.83, 0.99) and T2D (0.90; 0.84, 0.96).

#### 3.3.3 Adverse Outcomes

**FIGURE 3:**
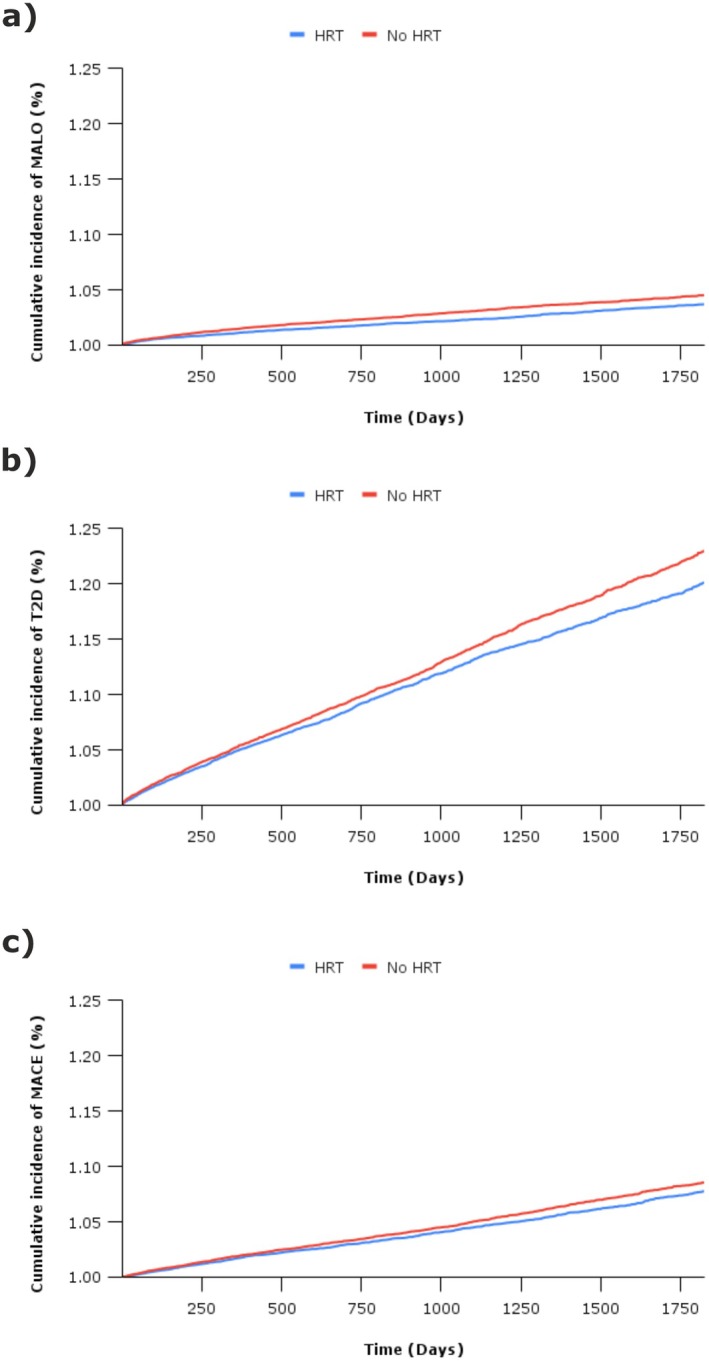
Cumulative incidence curves following Kaplan Meier survival analysis demonstrating the risk reduction in (a) MALO, major adverse liver outcomes, (b) T2D, type 2 diabetes, and (c) MACE, major adverse cardiovascular events, following treatment of peri‐menopausal symptoms in women with concomitant MASLD using HRT (versus no HRT).

**FIGURE 4:**
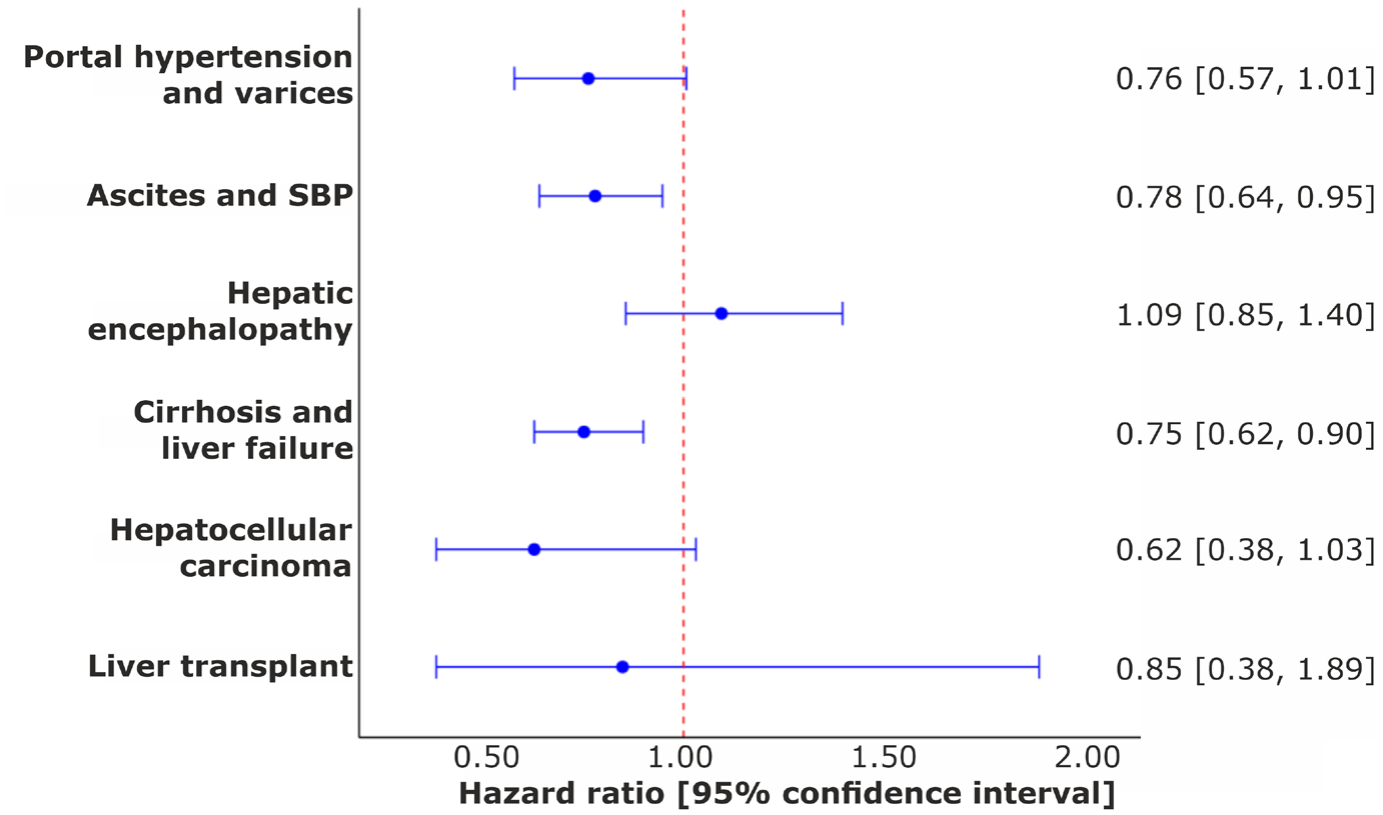
Forest plot of hazard ratios with 95% confidence intervals generated through Kaplan Meier survival analysis demonstrating the risk reduction in individual MALO endpoints, following treatment of peri‐menopausal MASLD with HRT (compared to no HRT).

Finally, HRT was not associated with any significantly risk of the adverse outcomes: breast and endometrial cancer, or VTE. In fact, HRT was associated with significant risk reduction in endometrial cancer (0.49; 0.40, 0.61). All numerical data is presented in Table 2, whilst survival curves are presented in Figure 3 and a forest plot in Figure 4.

**TABLE 2:** Major adverse liver outcomes as a composite measure with individual events in patients treated with HRTvs. no HRT.

	Sample size	Outcome (*n*)	5‐year survival (%)	Hazard ratio [95% confidence interval]	Log‐rank	*p*	*E*‐value
**Major adverse liver outcome (composite)**							
No HRT	21 639	625	95.7	Reference			
HRT	21 639	501	96.4	**0.80 [0.71, 0.90]**	13.9	< 0.01	1.81
**Individual major adverse liver outcome components**							
*Portal hypertension and varices*							
No HRT	21 639	113	99.2	Reference			
HRT	21 639	86	99.4	0.76 [0.58, 1.01]	3.7	0.06	1.00
*Ascites or spontaneous bacterial peritonitis*							
No HRT	21 639	225	98.4	Reference			
HRT	21 639	175	98.7	**0.78 [0.64, 0.95]**	6.3	0.01	1.88
*Hepatic encephalopathy*							
No HRT	21 639	122	99.1	Reference			
HRT	21 639	133	99.0	1.09 [0.86, 1.40]	0.5	0.48	1.00
*Hepatorenal/pulmonary syndrome* ^a^							
No HRT	21 639	NA	NA	Reference			
HRT	21 639	NA	NA	**NA**	NA	NA	1.00
*Cirrhosis or liver failure*							
No HRT	21 639	272	98.1	Reference			
HRT	21 639	204	98.6	**0.75 [0.63, 0.90]**	9.7	< 0.01	2.00
*Hepatocellular carcinoma*							
No HRT	21 639	40	99.7	Reference			
HRT	21 639	25	99.8	0.63 [0.38, 1.03]	3.5	0.06	1.00
*Liver transplant*							
No HRT	21 639	13	99.9	Reference			
HRT	21 639	11	99.9	0.85 [0.38, 1.89]	0.2	0.69	1.00
**Type 2 diabetes**							
No HRT	15 179	1897	81.3	Reference			
HRT	15 274	1713	83.2	**0.90 [0.84, 0.96]**	10.4	< 0.01	1.46
**Major adverse cardiovascular events (composite)**							
No HRT	20 089	992	92.1	Reference			
HRT	20 175	899	92.8	**0.90 [0.83, 0.99]**	5.0	0.03	1.46
**Adverse outcomes**							
*Breast cancer*							
No HRT	20 337	330	97.4	Reference			
HRT	20 883	321	97.7	0.90 [0.77, 1.05]	1.9	0.16	1.00
*Endometrial cancer*							
No HRT	21 639	265	98.5	Reference			
HRT	21 639	131	99.2	**0.49 [0.40, 0.61]**	45.4	< 0.01	3.50
*Venous thromboembolism*							
No HRT	21 639	330	99.7	Reference			
HRT	21 639	329	99.7	1.00 [0.86, 1.16]	< 0.1	0.96	1.00

*Note:* Bold values indicates statistical significance.

^a^ In TriNetX, any outcome with an event count < 10 is reported as < 10 rather than the true value in line with their confidentiality and data sharing agreement, and therefore unable to undergo survival analysis.

### 3.4 Sensitivity Analyses

Results for all sensitivity analyses are presented in Table S3.

#### 3.4.1 Degree of Overweight or Obesity

Among patients with mild‐to‐moderate obesity (BMI > or < 40 kg/m^2^), HRT was associated with significant risk reductions for MALO (0.80; 0.70, 0.92), T2D (0.89; 0.82, 0.97), but not MACE (0.93; 0.84, 1.04).

In patients with severe obesity, HRT was only associated with a reduced risk of T2D (0.81; 0.68, 0.96); MALO (0.85; 0.61, 1.18), and MACE (0.86; 0.68, 1.08) were not associated with significant risk reduction.

In lean MASLD (BMI < 30 kg/m^2^), HRT was only associated with significant risk reductions in T2D (0.82 [0.73, 0.92]).

#### 3.4.2 Oestrogen vs. Progesterone

Patients treated with oestrogen‐based HRT, without progesterone, were associated with reduced risks of MALO (0.83; 0.72, 0.95), T2D (0.85, 0.78; 0.92), and MACE (0.88; 0.78, 0.98), whereas patients treated with progesterone‐based HRT, without oestrogen, did not have significant risk reduction in any of MALO, T2D, or MACE.

#### 3.4.3 Geographical Location

In patients from the USA, HRT treatment remained associated with significant risk reduction for MALO (0.86; 0.76, 0.97) and T2D (0.85; 0.80, 0.91) but not MACE (0.92; 0.84, 1.01).

#### 3.4.4 Ethnicity

Patients of white ethnicity treated with HRT were associated with significant risk reduction in T2D (0.87 [0.81, 0.94]), whereas patients from ethnic minority backgrounds treated with HRT were not associated with risk reduction in any outcome.

#### 3.4.5 Refined MALO Definition

When portal hypertension and non‐bleeding varices were removed from the MALO definition, patients treated with HRT had significantly lower associated risk of T2D (0.87 [0.82, 0.92]) and MACE (0.91 [0.84, 0.98]). The associated risk of MALO became marginally insignificant (0.90 [0.79, 1.02]), although there were associated significant risk reductions in bleeding varices (0.79 [0.64, 0.96]), ascites and SBP (0.77 [0.65, 0.92]), and liver failure (0.85 [0.74, 0.97]).

#### 3.4.6 HRT Persistence

When HRT was continued for at least 6 months, the associated risk of T2D (0.81 [0.74, 0.88]) and MACE (0.85 [0.76, 0.96]) were reduced. The associated risk of MALO was insignificantly reduced (0.88 [0.76, 1.02]), although there were significant associated risk reductions in portal hypertension (0.61 [0.44, 0.834]), ascites and SBP (0.73 [0.56, 0.943]), and HCC (0.45 [0.21, 0.98]).

## 4 Discussion

In this first real‐world study regarding the use of HRT and major adverse liver outcomes, we show that peri‐menopausal women with pre‐existing MASLD who receive HRT, compared with those who do not, have a 20% lower risk of a major adverse liver outcome over a 5‐year period. This reduction is largely driven by lower rates of ascites, SBP, cirrhosis, and liver failure. Furthermore, there is an additional benefit of a 10% lower risk of T2D and MACE over the same period. The benefit in this at‐risk MASLD cohort was most strongly associated with oestrogen‐, but not progesterone‐based, HRT. The protective effect was more robust in those patients with mild to moderate obesity, and less so in those with lean MASLD. Although there is scarcity of data demonstrating the impact of HRT on prevalence and progression of hepatic and extra‐hepatic outcomes in patients with MASLD, our data is consistent with other observational data highlighting a beneficial metabolic benefit of HRT in peri‐menopausal women with MASLD.

### 4.1 Baseline Prevalence of MASLD According to Menopausal Status and HRT Use

In humans, data from cross‐sectional studies suggests that the frequency of MASLD is higher in postmenopausal than in premenopausal women [15, 16]. Data from the National Health and Nutrition Examination Survey (NHANES) III (1988–1994; U.S. population) provided early evidence of a potential protective effect of HRT on MASLD, with postmenopausal users showing a 31% lower odds of MASLD than non‐users [17]. Although an important early finding, the cross‐sectional design means residual confounding, particularly from T2D and dyslipidaemia, may have influenced the results. Supporting this, data from 251 Brazilian patients showed a lower prevalence of ultrasound‐confirmed MASLD in HRT users (26.4% vs. 39.9%) along with better liver biochemistry, whereas non‐users had greater metabolic dysfunction, including greater insulin resistance and a higher prevalence of metabolic syndrome [18]. However, a pooled cross‐sectional analysis of three U.S. studies in biopsy‐proven MASLD reported higher odds of lobular inflammation in HRT users compared with non‐users, although this association became marginal after adjustment for confounders. Rates of hepatocellular ballooning were similar between groups, and a follow‐up analysis suggested the signal was driven by progestogen rather than oestrogen. The study's interpretation is limited by referral, recall, and selection biases, and the lack of data on cumulative HRT exposure [19].

### 4.2 Progression or Regression of MASLD

Longitudinal evidence linking HRT to MASLD progression or regression is limited and inconsistent. In a post hoc analysis of a 6‐month trial in postmenopausal women with T2D (UK), oestradiol (1 mg) plus norethisterone (0.5 mg) led to greater reductions in liver enzymes (AST, ALT, ALP, GGT) than placebo. Higher baseline enzyme levels, suggestive of underlying MASLD, MASH, or fibrosis, were associated with larger improvements, indicating greater benefit in women with poorer baseline liver biochemistry [20]. Although the trial did not specifically enrol patients with MASLD, the high prevalence of MASLD in T2D (~70%) indicates that many participants were likely affected [21]. This trial also did not include imaging or biopsy measures of liver fat or fibrosis. However, a prospective 12‐month study of 368 postmenopausal women found that HRT led to MASLD resolution on ultrasound, with benefits confined to the transdermal group, which saw a 7% reduction in steatosis. The lack of metabolic changes in this group suggests a mechanism related to bypassing first‐pass hepatic metabolism: oral HRT increased triglycerides, whereas transdermal therapy had neutral or favourable lipid effects, aligning with its lower MASLD risk [22]. Given the limitations of the TriNetX platform in respect to medication coding, we were unable to perform stratified analyses based on the administration route of HRT. In contrast, results arise from a study in 1829 Japanese patients, which found no statistically significant difference in MASLD prevalence between patients treated with or not treated with (5.3 vs. 6.1%), HRT [23]; acknowledging the atypically low prevalence of MASLD in this cohort.

### 4.3 Metabolic Benefits

A retrospective cohort study using the TriNetX (USA) network found that among 6566 peri‐menopausal patients with pre‐diabetes, HRT was associated with a 31% lower risk of progression to T2D over 20 years, with greater protection in women with overweight than those with obesity [24]. This is supported by a separate retrospective study of 595 peri‐ and postmenopausal women from Zhejiang Province, in which HRT improved glucose and lipid metabolism, including reductions in fasting insulin, HOMA‐IR, and fasting glucose, as well as lower LDL and total cholesterol compared with baseline [25].

### 4.4 Cardiovascular Benefits

To our knowledge, no study has examined the impact of HRT on cardiovascular disease specifically in patients with MASLD. However, substantial evidence exists in the general population. A clinical trial showed that oestradiol therapy slowed progression of subclinical atherosclerosis, measured by carotid intima–media thickness, when initiated within 6 years of menopause, but not when started 10 or more years later [26]. Supporting this, observational data reports a 40%–50% reduced risk of coronary heart disease (CHD) following treatment with HRT [27]. In contrast, the Women's Health Initiative (WHI) randomised trial found increased CHD risk with combined oestrogen–progestin therapy, concluding that overall risks outweighed benefits over 5.6 years [28]. A subsequent combined analysis of the WHI trial and observational cohort showed that much of this discrepancy was explained by confounding and, critically, by timing of initiation. Early HRT use carries a short‐term increase in CHD, stroke and venous thromboembolism risk, whereas longer‐term use when initiated closer to menopause may yield neutral or favourable cardiovascular outcomes; supporting the ‘timing hypothesis’ [29]. This is consistent with our study, in which peri‐menopausal women with MASLD treated with HRT had a significantly lower risk of MACE over 5 years. Our data extend the timing hypothesis to women with MASLD, an understudied population with elevated cardiometabolic burden, highlighting the potential for appropriately timed HRT to mitigate both hepatic and cardiovascular complications.

### 4.5 Mechanisms

Extensive experimental and clinical evidence supports a protective role for oestrogen in liver health. Physiological oestrogen concentrations suppress pro‐inflammatory cytokines (IL‐1, IL‐6, TNF‐α), whereas menopause and the associated decline in ovarian hormones are linked to increased cytokine production [30]. Oestrogen deficiency is also associated with more rapid fibrosis progression in chronic liver disease [31], while experimental studies show that oestrogen inhibits hepatic stellate cell activation and reduces collagen deposition [32]. In addition, ERα signalling improves hepatocyte mitochondrial function, lowering hepatic lipid accumulation and insulin resistance [33].

Across the female lifespan, oestrogen is central to metabolic homeostasis. Higher 17β‐oestradiol levels in the reproductive years correlate with lower MASLD prevalence, whereas later menarche and longer reproductive lifespan are associated with reduced risk. The paradoxical association between early menarche and higher long‐term MASLD risk likely reflects underlying childhood adiposity, metabolic dysfunction and premature activation of the hypothalamic–pituitary–gonadal axis, rather than a detrimental effect of oestrogen itself. Oestrogen also supports metabolic health by promoting favourable fat distribution, enhancing insulin sensitivity, improving mitochondrial oxidative capacity and limiting hepatic de novo lipogenesis [34]. During the menopausal transition, falling oestrogen levels drive increases in visceral adiposity, inflammation and adipose tissue fibrosis [8], alongside shifts in ERα/ERβ expression that impair metabolic signalling and predispose to steatosis and fibrogenesis [34] (Figure S2).

Consequently, postmenopausal women have a markedly higher prevalence of MASLD, with severity tracking the degree and duration of oestrogen deficiency. Restoring physiological oestrogen levels through HRT may therefore counteract these changes, improving adipose tissue insulin sensitivity, reducing hepatic lipid accumulation and slowing fibrogenesis, consistent with the hepatic benefits observed in our cohort.

### 4.6 Benefits of HRT in Other Aetiologies of Liver Disease

HRT has also shown favourable effects in non‐MASLD liver conditions. In a retrospective cohort of 6160 women with steatotic liver disease secondary to hepatitis B from Taiwan's National Health Insurance Research Database, HRT use was associated with a 50% lower risk of HCC and a 51% reduction in all‐cause mortality over 18 years. Notably, the greatest protection occurred with 12–36 months of therapy, with benefits diminishing at longer durations [35].

### 4.7 Clinical Implications

Our findings suggest that HRT, particularly oestrogen‐based therapy, may offer meaningful protection against both hepatic and extra‐hepatic complications in peri‐menopausal women with MASLD, without increasing recognised risks such as breast or endometrial cancer or VTE. Although current NICE guidance recommends offering HRT for menopausal symptoms through shared decision‐making, accounting for cardiovascular, oncological and thrombotic risks, it does not consider liver health or MASLD [10]. Given growing evidence linking oestrogen deficiency, menopause and MASLD progression, future guidelines should incorporate liver and cardiometabolic risk into HRT initiation discussions. By reducing progression to MALO, T2D and MACE, HRT may represent a modifiable therapeutic option in this high‐risk population. These findings raise important questions, including whether selected peri‐ or post‐menopausal women with MASLD should be considered for HRT irrespective of symptom burden, and whether MASLD screening should form part of routine menopause assessment. Clinical decisions should remain individualised, considering metabolic profile, adiposity, liver disease severity, menopausal symptoms, and the formulation and route of HRT. Finally, our real‐world evidence highlights the need for well‐designed longitudinal and interventional studies, such as the ongoing trial NCT04833140, to determine the optimal use of HRT in MASLD [36].

### 4.8 Limitations

We acknowledge multiple limitations in the data set. First, in real‐world data comparisons are neither randomised nor controlled. Second, there is potential for a lack of data completeness resulting from data being extracted from EHRs of an administrative database; data may not be recorded by the HCO, or other data recorded in free text which we are unable to extrapolate. Specifically, the TriNetX platform does not allow assessment of the timing or recency of individual measurements prior to index date (e.g., how long before cohort entry laboratory or anthropometric values were last recorded), limiting our ability to formally assess data completeness at the variable level. In addition, should participants move between HCOs, it is possible that some of their data may not be available to us as one or more of their HCOs may not form part of the global collaborative network. Third, information concerning dosage and route of HRT administration was not available to us, and therefore we were unable to comment on the dose‐, or route‐, dependent relationship of HRT on incident MALO. Fourth, as with any large database study, residual confounding remains possible. Furthermore, alcohol consumption is poorly coded on TriNetX and therefore we are unable to report on patients who may living with MetALD (mixed MASLD and moderate alcohol consumption). To mitigate as much as possible against this, our PSM model included available biochemical markers of liver disease (transaminases, bilirubin, prothrombin time) which are associated with the degree of alcohol intake [37]. Similarly, although we can be confident of a MASLD diagnosis using our approach, we are unable to accurately provide an accurate liver risk stratification since we cannot characterise the histological or biochemical severity of MASLD; assessing steatosis, fibroinflammation or fibrosis, in the patients included in this study. Such data are not routinely collected in clinical practice and are therefore unavailable within the TriNetX platform, a limitation of many real‐world evidence studies. Given that fibrosis stage is the strongest predictor of liver‐related outcomes, this represents the most important potential source of residual confounding. As a result, we cannot determine whether differences in baseline disease stage contributed to the observed differential protection, particularly among individuals living with obesity. Although our propensity score model incorporated several variables that correlate with advanced fibrosis risk, including age, liver biochemistry, platelet count and cardiometabolic comorbidity, these serve only as indirect proxies rather than direct measures of disease stage. It is therefore likely that a proportion of patients may have been living with asymptomatic or undiagnosed advanced liver disease, including fibrosis or cirrhosis, which could reduce responsiveness to pharmacological interventions and partially explain the heterogeneity of treatment effects observed. Further, although differential body weight changes over the course of follow up could plausibly mediate some association between HRT and liver or cardiometabolic outcomes, reliable, cohort‐matched, longitudinal assessment of BMI change is not feasible within TriNetX, and therefore such analyses would be potentially misleading. However, we ensured appropriate matching on entry to the study. Baseline BMI was well balanced between groups after propensity score matching (33.2 vs. 33.5 kg/m^2^; standardised mean difference 0.05), making pre‐existing differences in adiposity an unlikely explanation for the observed associations. While differential weight trajectories cannot be excluded, available evidence suggests HRT may only modestly attenuate peri‐menopausal increases in adiposity, with heterogenous and small or non‐significant effects on absolute body weight [38, 39]. These considerations indicate that reduced weight gain alone with HRT use is unlikely to account for the consistent reductions in MALO, T2D and MACE observed. To indicate the potential impact of unmeasured confounding, we calculated *E*‐values to quantify the minimum strength of association that an unmeasured confounder would need to have with both the exposure and the outcome to fully explain away the observed associations [13]. Finally, in our statistical approach, cumulative incidence was estimated using the Kaplan–Meier method, which may overestimate risk in the presence of competing risks such as non–MALO death, compared other estimations (i.e., Aalen–Johansen estimator). Unfortunately, the TriNetX platform performs analyses in situ and does not allow users to alter the statistical approach beyond the Kaplan–Meier method. As such, we acknowledge this as a methodological limitation of our study.

## 5 Conclusion

Treatment of peri‐menopausal symptoms with HRT, particularly estrogen‐based HRT, in patients with pre‐existing MASLD, is associated with a lower risk of major liver and cardiometabolic disease. These findings should prompt further basic science research and clinical trials to address this important transition point in the life course of women.

## Author Contributions

Alex E. Henney was involved in project conception, data collection and analysis, and manuscript preparation. Jo Wilson was involved in manuscript preparation. David R. Riley provided statistical oversight and was involved in manuscript preparation. Uazman Alam provided senior supervision and was involved in manuscript preparation. Daniel J. Cuthbertson provided senior supervision and was involved in manuscript preparation.

## Funding

The authors have nothing to report.

## Conflicts of Interest

D.J.C. has received investigator‐initiated grants from Astra Zeneca and Novo Nordisk, support for education from Perspectum, financial remuneration for consultancy work from pharmaceutical company consultation (Madrigal, Roche) with all payments made to the University of Liverpool. He also serves as the Topic Advisor for Type 2 Diabetes medications for The National Institute for Health and Care Excellence (NICE), UK. U.A. has received honoraria from Procter & Gamble, Viatris, Grunenthal and Sanofi for educational meetings and funding for attendance to an educational meeting from Diiachi Sankyo. U.A. has also received investigator‐led funding by Procter & Gamble and is a council member of the Royal Society of Medicine's Vascular, Lipid & Metabolic Medicine Section. All other authors declare that there are no financial relationships or activities that might bias, or be perceived to bias, their contribution to this manuscript.

## Supporting information


**Data S1:** liv70562‐sup‐0001‐Supinfo1.docx.

## Data Availability

The data that support the findings of this study are available from TriNetX LLC, https://trinetx.com/, but third‐party restrictions apply to the availability of these data. The data were used under licence for this study with restrictions that do not allow for the data to be redistributed or made publicly available. However, for accredited researchers, the TriNetX data are available for licensing at TriNetX LLC. Data access may require a data sharing agreement and may incur data access fees. Data used in the generation of this paper were collected from the global TriNetX network and local data at LUHFT were not used.
