# Supplementary material for: Effect of Hormone Replacement Therapy on Liver and Cardiometabolic Outcomes in Peri‐Menopausal MASLD
Source: Liver Int. 2026 Feb 23;46(4):e70562. doi: 10.1111/liv.70562 (PMC12929706; doi:10.1111/liv.70562)
Supplement: Supplementary file 1 — Data S1: liv70562‐sup‐0001‐Supinfo1.docx. [file LIV-46-0-s001.docx]

**Impact**

**Supplementary Material** The impact of hormone replacement therapy on major adverse liver outcomes in peri-menopausal patients with metabolic dysfunction-associated steatotic liver disease: real-world evidence

**Table of contents**

- **Pages 2-6** Protocol
- **Pages 7-8** Target trial specification and emulation table (Supplementary Material Table 1)
- **Pages 8-9** Supplementary Material Table 2 (Definitions for all baseline diagnoses, covariates, and outcomes)
- **Page 10** Supplementary Material Figure 1 (Propensity score density curves for the four target trial emulations)
- **Page 11** Supplementary Material Tabe 3 (Results following stratified analyses)
- **Page 12** Supplementary Material Figure 2 (Mechanistic figure demonstrating the protective role oestrogen in liver fat accumulation)

**Protocol**

**1. Title**

- The impact of hormone replacement therapy on major adverse liver outcomes in peri-menopausal patients with metabolic dysfunction-associated steatotic liver disease: real-world evidence

**2. Research Question and Objectives**

- **Research Question**: Can treatment of peri-menopausal symptoms with hormone replacement therapy (HRT) reduce the incidence of major adverse liver outcomes (MALO) in patients with metabolic dysfunction-associated steatotic liver disease (MASLD)?
- **Primary Outcome**: A composite of MALO: portal hypertension, gastric and oesophageal varices, ascites, spontaneous bacterial peritonitis, hepatic encephalopathy, hepatorenal and hepatopulmonary syndrome, liver cirrhosis, liver failure, hepatocellular carcinoma, liver transplant.
- **Secondary Objectives**: The individual MALO endpoints, type 2 diabetes (T2D) and major adverse cardiovascular events (MACE).

**3. Study Design**

- Target Trial Emulation using retrospective longitudinal data from TriNetX.
- **Study Period**: Any time following data being available in the TriNetX platform.
- **Population**: Adult peri-menopausal patients (40-65 years, with ICD-10 coding for the menopause) with pre-existing MASLD (ICD-codes for steatotic liver disease, or positive modified hepatic steatosis index, with at least one feature of the metabolic syndrome (hypertension, dyslipidaemia, obesity, insulin resistance), and all other causes of chronic liver disease excluded).

**4. Eligibility Criteria**

- **Inclusion Criteria**:
  - Medical encounter with a health care organisation with an agreement for data sharing the TriNetX Global Collaborative Network.
  - Diagnosis of menopause (defined using: **i)** age between 40-65 years, and/or **ii)** ICD-10 code N95 or Z78.0.
  - Diagnosis of MASLD (defined using: **i)** ICD-code K76.0 for steatotic liver disease, or K75.81 for non-alcoholic steatohepatitis, or **ii)** positive modified hepatic steatosis index (age ≥50 years, alanine aminotransferase ≥30 u/L AND body mass index (BMI) ≥30 kg/m^2^), with at least one feature of the metabolic syndrome (hypertension (defined using: **i)** ICD-10 code I10, **ii)** systolic blood pressure ≥130 mmHg or diastolic blood pressure ≥85 mmHg, or **iii)** treatment with anti-hypertensive medication), dyslipidaemia (defined using: **i)** ICD-10 code E78, **ii)** triglycerides ≥150 mg/dL, or high density lipoprotein cholesterol <50 mg/dL, or **iii)** lipid lowering therapy), obesity (defined using: **i)** ICD-10 code E66, or **ii)** BMI ≥27 kg/m^2^), or insulin resistance (defined using: **i)** ICD-10 code E11, R73.01, R73.02, or R73.03, **ii)** HbA1c ≥6.5%, or **iii)** treatment with glucose lowering therapy)), and all other causes of chronic liver disease excluded).
  - Treatment of peri-menopausal symptoms with hormone replacement therapy (oestrogen and/or progesterone) or no treatment.
- **Exclusion Criteria**:
  - Diagnosis of any other cause of chronic liver disease (alcohol-use disorder (ICD-10 code F10), alcohol-related liver disease (K70), viral hepatitis (B15-B19), haemochromatosis (E83.11), Wilson’s disease (E83.01), primary sclerosing cholangitis (K83.01), primary biliary cirrhosis (K74.3), toxic liver disease (K71)).
  - Diagnosis of a MALO endpoint prior to the index event.
  - The reference arm could never have been prescribed HRT.

**5. Interventions**

- **Treatment Group**: Treatment with HRT (one, or both, of oestrogen and progesterone).
- **Comparison Group**: No treatment with HRT.

**6. Assignment of Interventions**

- **Definition of Initiation**: Initiation of HRT, defined as the first time the drug is coded on prescription records.
- **Baseline Definition**: The date of initiation of HRT for the treatment arm, and the date of diagnosis of the menopause for the reference arm.

**7. Follow-Up**

- **Start of Follow-Up**: The index event will be the date of initiation of HRT for the treatment arm, and the date of diagnosis of the menopause for the reference arm.
- **End of Follow-Up**: Maximum follow-up of 5 years. Patients will be censored if they receive coding for the outcome of interest, patient’s last known fact date, or end of the time-window for analysis.

**8. Outcomes**

- **Primary Outcome**: The MALO composite.
- **Secondary Outcomes**:
  - Individual MALO endpoints.
  - T2D.
  - MACE.

**9. Data Sources**

- We aim to explicitly emulate the target trials described using data and built-in analytic functions on the TriNetX Analytics platform. TriNetX (LLC, Cambridge, MA, USA) is a global federated health research network that has access to both inpatient and outpatient electronic medical records from health care organisations internationally; largely secondary, and tertiary care providers in North America and Western Europe. This analysis will be conducted using the Global Collaborative Network, which contains data from ~180 million patients (from over 150 HCOs) with access to diagnoses, procedures, medications, laboratory values and genomic information worldwide. The built-in analytics within the TriNetX Analytic platform will analyse patient-level data; however, only population-level results will be reported to the research team. TriNetX data are HIPAA (Health Insurance Portability and Accountability Act) de-identified and access to protected health information is not allowed. Therefore, there is no risk for protected health information disclosure, and Institutional Review Board review was not required. Further details on the network have been described by Palchuk, M.B., et al (A global federated real-world data and analytics platform for research. JAMIA Open, 2023. 6(2): p. ooad035).

**10. Confounding and Bias Control**

- **Confounding Variables**: Cohorts will be propensity score matched (PSM), in a 1:1 ratio using greedy nearest neighbour matching, for **i)** sociodemographic variables: age, sex, ethnicity, smoking, socioeconomic status (problems relating to education and literacy, employment, housing, and psychosocial circumstances), **ii)** comorbidities: cardiovascular disease (ischaemic heart disease (IHD), cerebrovascular accident (CVA), hypertension and dyslipidaemia), type 2 diabetes, and thyroid disease, and cancer, **iii)** anthropometrics: BMI, **iv)** biochemistry: HbA1c, platelets, albumin, alanine (ALT) and aspartate aminotransferase (AST), bilirubin and prothrombin time, and **v)** medication: glucose lowering therapy, lipid-lowering therapy, anti-coagulation, anti-platelets, steroids and anti-hypertensives.
- **Strategies to Address Confounding**:
  - Propensity score matching.
  - Emulation of target trials.
- **Handling Missing Data**: TriNetX will perform only complete case-analysis.

**11. Statistical Analysis Plan**

- **Analysis Population**: Intention-to-treat, per-protocol.
- **Primary Analysis**: Survival analysis.
- **Sensitivity Analyses**: We will perform the following sensitivity analyses:
- Geographical location; refining the search to the US Collaborative Network only.
- Severity of obesity; mild-to-moderate obesity (BMI between 27-40 kg/m^2^), and severe obesity (BMI ≥40 kg/m^2^).
- Type of HRT; oestrogen or progesterone.
- Calculate E-values, representing the minimum strength of association on the HR scale that an unmeasured confounder would need to have with both the exposure (treatment arm) and the outcome, conditional on the measured confounders, to explain away the observed association; HR+√[HR×(HR-1)].

**12. Assumptions and Limitations**

- Firstly, these will be real-world data and therefore do not provide randomised or controlled comparisons.
- Secondly, in data extracted from electronic health records in an administrative database, there is potential for a lack of data completeness. This is amplified in the use of open circuit databases like TriNetX where it is possible that a patient may move outside of the HCO and therefore longitudinal data is lost. TriNetX will exclude missing values from any relevant analysis, but it does not provide imputation or any other statistical technique.
- Thirdly, residual bias confounding remains possible despite PSM with potential confounding variables, such as accurate alcohol consumption levels at index, and smoking, both being poorly coded. To address this, we will PSM for liver enzymes at baseline as a biochemical surrogate for alcohol consumption/baseline liver disease severity and will further attempt to reduce the risk of unidentified residual confounding through calculation of E-values as a quantitative bias analysis to assist readers in the interpretation of the strength of our results.

**13. Ethical Considerations**

- TriNetX data are HIPAA (Health Insurance Portability and Accountability Act) de-identified and access to protected health information is not allowed. Therefore, there is no risk for protected health information disclosure, and Institutional Review Board review was not required

**14. Dissemination Plan**

- Plans to publish findings in peer-reviewed journals and present at conferences.

| **Protocol component** | **Target Trial Specification** | **Target Trial Emulation** |
| --- | --- | --- |
| **Eligibility criteria** | **Inclusion criteria**   - Adult per-menopausal (40-65 years) patients with pre-existing MASLD defined using ultrasound; diagnosed with MASLD before or at baseline - Eligible for treatment with HRT under prevailing clinical practice guidelines   **Exclusion criteria**   - Diagnosis of another cause of chronic liver disease - Diagnosis of a major adverse liver outcome endpoint prior to study start - Contra-indication to HRT - Pregnancy | Same as for Target Trial Specification except for we cannot use ultrasonography to detect cases of previously undiagnosed MASLD.  We used data from the TriNetX platform between May 2022–Nov 2023. Patients treated with DPP4i mimicked our placebo group from the Target Trial Specification. |
| **Treatment strategies** | Participants were randomised to receive HRT or placebo. | Initiation of HRT vs no HRT. There is no suitable ‘reference’ treatment that can act as the placebo in this target trial, so no treatment will act as placebo arm. Index event is the day of treatment prescription. Intention to treat analysis at assignment. Follow-up up to 5 years. |
| **Treatment assignment** | Participants allocated (1:1) to HRT or placebo using a computer-generated random sequence. All participants, investigators, and the sponsor, were blinded; only a very limited support team had access to randomisation data. | 1:1 propensity-score matching (nearest-neighbour greedy, caliper 0.1 SD). Balance assessed with SMD (<0.1 threshold in practice). Analyses on matched cohorts. |
| **Outcomes** | **Primary**   - Incident in major adverse liver outcome | **Primary**   - Incident major adverse liver outcome   **Secondary**   - Individual liver outcome endpoints, type 2 diabetes, major adverse cardiovascular events. |
| **Follow-up** | Baseline = first administration of HRT; follow to 56 weeks + 4-week safety or earlier if outcome/discontinuation; rescue allowed per protocol. | Baseline = day after index prescription; follow to first of outcome, death, loss to follow-up, or 5 years. |
| **Causal contrast** | ICH E9 (R1) treatment-regimen estimand (policy, regardless of discontinuation/rescue) and efficacy estimand (hypothetical, if all adhered and no rescue). | Observational analogue of ITT for HRT vs no HRT. Sensitivity: geographical location, degree of obesity, type of HRT. |
| **Analysis** | Graphical testing to control type I error (α=0.05 within estimand); multiple imputation (treatment-regimen estimand); MMRM for efficacy estimand; SAS 9.4. | Kaplan–Meier for cumulative incidence; Cox models for HRs with 95% CIs in matched cohorts; TriNetX built-in analytics (R survival v3.2-3). E-values computed for unmeasured confounding; STROBE followed. |

**Supplementary Material Table 1** Target trial specification and emulation (current observational study). The left column describes the protocol components of the hypotehtical randomised controlled trial. The right column describes how each component was emulated using electronic health records from the TriNetX US Collaborative Network to evaluate HRT vs. no HRT for prevention of incident major adverse liver outcomes. “Causal contrasts” refer to the policy/intention-to-treat estimand (assignment regardless of adherence or rescue therapy) and the hypothetical/per-protocol estimand (if all patients adhered without rescue).

| **Diagnosis** | **ICD-10, CPT or SNOMED code** |
| --- | --- |
| **Inclusion criteria** | |
| Menopause | N95, Z78.0 |
| Steatotic liver disease | K76.0, K75.81 (or age ≥50 years, ALT ≥30 u/L, and BMI ≥30 kg/m^2^) |
| Obesity | E66.9 (or BMI >27 kg/m^2^) |
| Insulin resistance | E11, R73.01, R73.02, or R73.03 (or HbA1c ≥6.5%, treatment with glucose lowering therapy) |
| Hypertension | I10 (or blood pressure ≥130/85 mmHg, prescription of anti-hypertensive medication) |
| Dyslipidaemia | E78 (or triglycerides >150 mg/dL, HDL cholesterol <50 mg/dL, prescription of lipid lowering therapy) |
| **Exclusion criteria** | |
| Alcohol-related disorders | F10 |
| Alcohol-related liver disease | K70 |
| Viral hepatitis | B15-B19 |
| Haemochromatosis | E83.11 |
| Wilson’s disease | E83.01 |
| Primary sclerosing cholangitis | K83.0 |
| Primary biliary cholangitis | K74.3 |
| Toxic liver disease | K71 |
| Autoimmune hepatitis | K75.4 |
| Portal hypertension | K76.6 |
| Ascites | R18 |
| Spontaneous bacterial peritonitis | K65.2 |
| Hepatic encephalopathy | K76.82 |
| Hepatorenal syndrome | K76.7 |
| Hepatopulmonary syndrome | K76.81 |
| Oesophageal varices | I85 |
| Gastric varices | I86.4 |
| Liver failure | K72.9 |
| Liver cirrhosis | K74.6 |
| Liver transplant | 47133, 47135, 47141, 47142, 47143, 47144, 47145, 47146, 47147, 18027006, 27280000, 426356008, 853761000000103, 213153001, 235911006, 235912004, 737297006, 96601000119101, Z94.4, T86.4, T86.41, T86.42, T86.49, 0FY00Z0, 0FY00Z1, 0FY00Z2, 0FY00Z3 |
| Hepatocellular carcinoma | C22 |
| **Covariates** | |
| Ischaemic heart disease | I20-I25 |
| Cerebrovascular disease | I60-I69 |
| Peripheral vascular disease | I73 |
| Heart failure | I50 |
| Cancer | C00-D49 |
| Disorders of the thyroid gland | E00-E07 |
| Nicotine dependence | F17.2 |
| Socioeconomic hazards | Z55-Z65 |
| **Outcome** | |
| Major adverse liver outcomes | As exclusion criteria |
| Type 2 diabetes | E11 |
| Major adverse cardiovascular events | Z86.74, I21-I23, I63, I50 |
| Breast cancer | C50 |
| Endometrial cancer | C54 |
| Venous thromboembolism | I84 |

**Supplementary Material Table 2** Definitions for all baseline diagnoses, covariates, and outcomes.


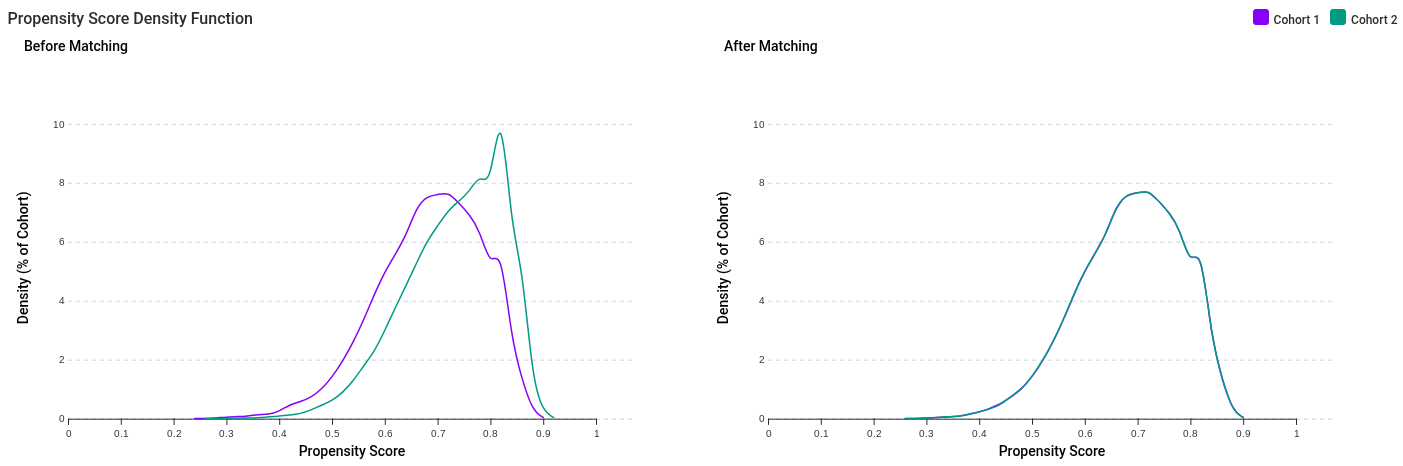


**Supplementary Material Figure 1** Density curves to demonstrate covariate density pre- and post-propensity score matching between the hormone replacement therapy (HRT) (cohort 1, purple), and no HRT (cohort 2, green), arms.

| **Stratification** | **Hazard ratio [95% confidence interval]** |
| --- | --- |
| **Geographical location** | |
| ***Restricted to USA*** | |
| Major adverse liver outcomes | 0.86 [0.76, 0.97] |
| Type 2 diabetes | 0.85 [0.80, 0.91] |
| Major adverse cardiovascular events | 0.92 [0.84, 1.01] |
| **Degree of overweight or obesity** | |
| ***BMI >40 kg/m^2^*** | |
| Major adverse liver outcomes | 0.85 [0.61, 1.18] |
| Type 2 diabetes | 0.81 [0.68, 0.96] |
| Major adverse cardiovascular events | 0.86 [0.68, 1.08] |
| ***BMI <40 kg/m^2^*** | |
| Major adverse liver outcomes | 0.80 [0.70, 0.92] |
| Type 2 diabetes | 0.89 [0.80, 0.97] |
| Major adverse cardiovascular events | 0.93 [0.84, 1.04] |
| ***BMI <30 kg/m^2^*** | |
| Major adverse liver outcomes | 0.91 [0.77, 1.07] |
| Type 2 diabetes | 0.82 [0.73, 0.92] |
| Major adverse cardiovascular events | 0.97 [0.82, 1.13] |
| **Type of hormone replacement therapy** | |
| ***Oestrogen*** | |
| Major adverse liver outcomes | 0.83 [0.72, 0.95] |
| Type 2 diabetes | 0.85 [0.78, 0.92] |
| Major adverse cardiovascular events | 0.88 [0.78,0.98] |
| ***Progesterone*** | |
| Major adverse liver outcomes | 1.21 [0.86, 1.70] |
| Type 2 diabetes | 1.05 [0.88, 1.23] |
| Major adverse cardiovascular events | 1.18 [0.92, 1.52] |
| **Ethnicity** | |
| ***White*** | |
| Major adverse liver outcomes | 0.90 [0.81, 1.02] |
| Type 2 diabetes | 0.87 [0.81, 0.94] |
| Major adverse cardiovascular events | 0.93 [0.84, 1.02] |
| ***Ethnic minority groups*** | |
| Major adverse liver outcomes | 0.86 [0.71, 1.03] |
| Type 2 diabetes | 0.93 [0.83, 1.03] |
| Major adverse cardiovascular events | 0.88 [0.74, 1.03] |
| **Refined major adverse liver outcomes definition** | |
| Major adverse liver outcomes | 0.90 [0.79, 1.02] |
| Type 2 diabetes | 0.87 [0.82, 0.92] |
| Major adverse cardiovascular events | 0.91 [0.84, 0.98] |
| **Hormone replacement therapy persistence** | |
| Major adverse liver outcomes | 0.88 [0.76, 1.02] |
| Type 2 diabetes | 0.81 [0.74, 0.88] |
| Major adverse cardiovascular events | 0.85 [0.76, 0.96] |

**Supplementary Material Table 3** Results following stratified analyses.


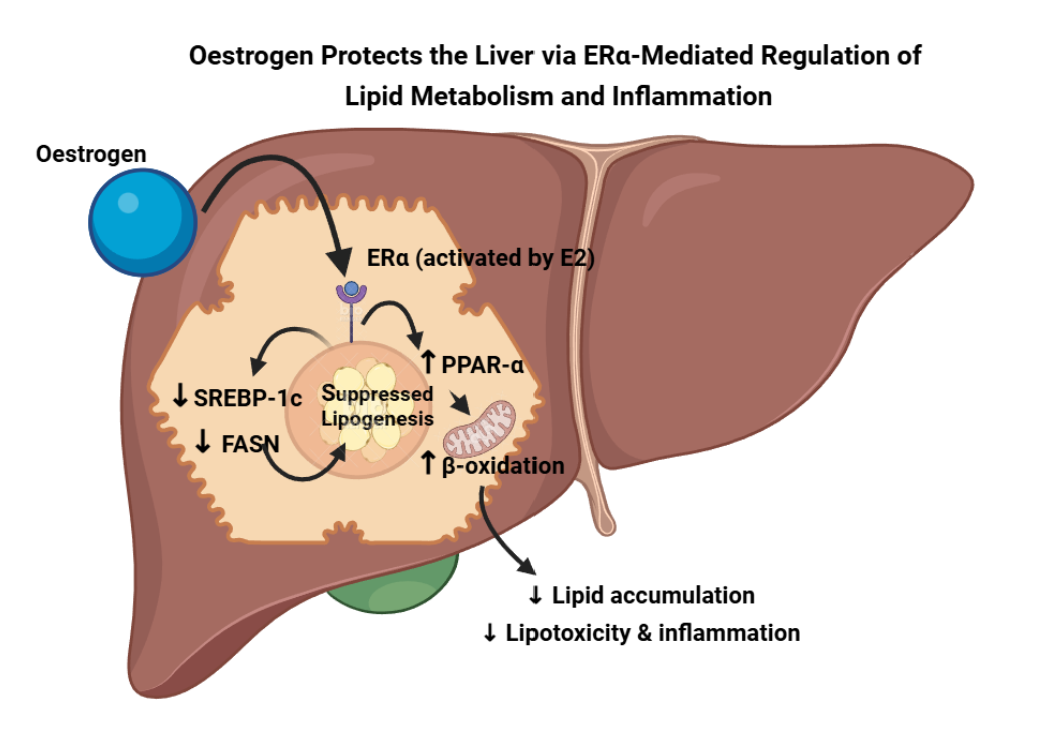


**Supplementary Material Figure 2** Mechanistic illustration demonstrating how oestrogen protects the liver via oestrogen-receptor alpha mediated regulation of lipid metabolism and inflammation.

**Abbreviations** *ERa, oestrogen-receptor alpha; SREBP-1c, sterol regulatory element-binding protein 1c; FASN, fatty acid synthase gene; PPAR-a, peroxisome proliferator-activated receptor alpha.*
